# Unusual Spread of Renal Cell Carcinoma to the Clivus with Cranial Nerve Deficit

**DOI:** 10.1155/2016/9184501

**Published:** 2016-03-15

**Authors:** Jerome Okudo, Nwabundo Anusim

**Affiliations:** ^1^School of Public Health, University of Texas, 1200 Pressler Street, Houston, TX 77030, USA; ^2^Department of Medicine, Saint Joseph Regional Medical Center, 5215 Holy Cross Parkway, Mishawaka, IN 46545, USA

## Abstract

Renal cell carcinoma (RCC) has unusual presentation affecting elderly males with a smoking history. The incidence of RCC varies while the incidence of spread of RCC to the clivus is rare. The typicality of RCC presentation includes hematuria, flank pain, and a palpable flank mass; however, RCC can also present with clival metastasis. The unique path of the abducens nerve in the clivus makes it susceptible to damage in metastasis. We report a case of a 54-year-old African American female that was evaluated for back pain, weakness, numbness, and tingling of bilateral lower extremities and subsequently disconjugate gaze and diplopia. Brain MRI confirmed metastasis to the clivus. She was started on radiotherapy and was planned for chemotherapy and transfer to a nursing home. When a patient presents with sudden unusual cranial nerve pathology, the possibility of metastatic RCC should be sought.

## 1. Introduction

The involvement of the clivus due to tumor metastasis is a rarity in clinical practice. Very limited literature is available on spread of RCC to the clivus [1, 2]. RCC accounts for less than 5% of all diagnosed malignancies in adults [3]. 25% of RCC are known to have spread at the time at which the diagnosis of malignancy is confirmed [3]. Metastasis from RCC usually occurs to the lungs in half of the cases; other areas include bone, lymph node, and the brain [3]. Cancer spread to the clivus is becoming better known; however, very few cases of spread from RCC have been reported [1, 3]. Considering the location of the clivus, metastasis to this area can present with multiple cranial nerve pathologies such as diplopia.

## 2. Case Presentation

A 54-year-old African American female with a history of hypertension presented to the emergency department with back pain of three-week duration. The pain radiated to the bilateral lower extremities with associated weakness, numbness, and tingling sensation, which was worse on the left lower extremity. She was diagnosed with sciatica, given a prescription of methocarbamol, and discharged home. She presented a few days later with constipation and was given stool softeners. A few days later, she presented with complaints of urinary retention and worsening back pain. Her examination revealed hyperactive tendons, no ankle clonus and complete loss of sensation, diminished proprioception in the right lower extremity, and proximal weakness in both lower extremities. The left and right iliopsoas muscle were 3/5 and 2/5, respectively; left and right quadriceps were 3/5 and 2/5, respectively; there were 4+/5 and 4/5 on dorsiflexion and plantar flexion was 4+ bilaterally. While investigations were ongoing for her symptoms, she complained of diplopia and physical examination revealed disconjugate gaze.

**Figure 1:**
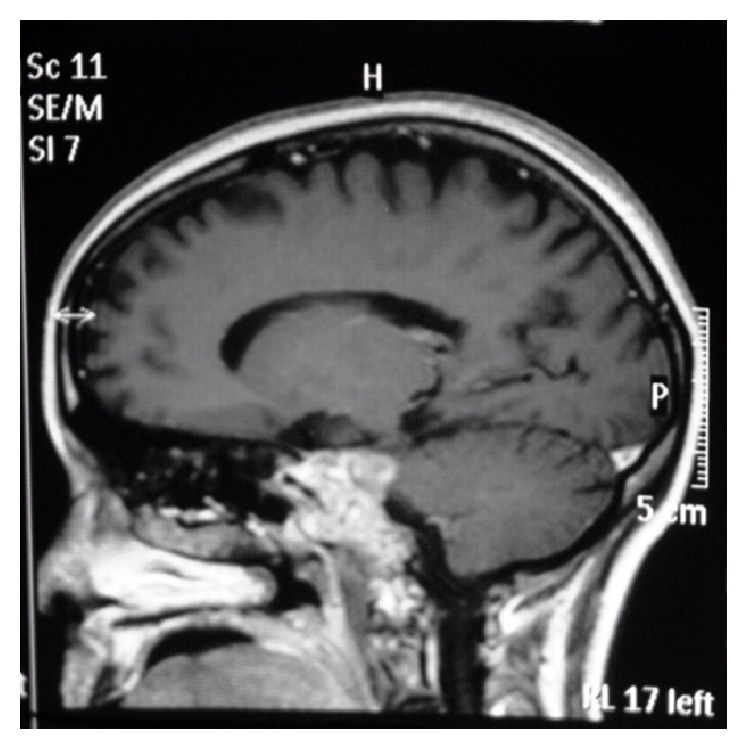
Sagittal section of the brain without contrast showing the clival lesion.

**Figure 2 fig2:**
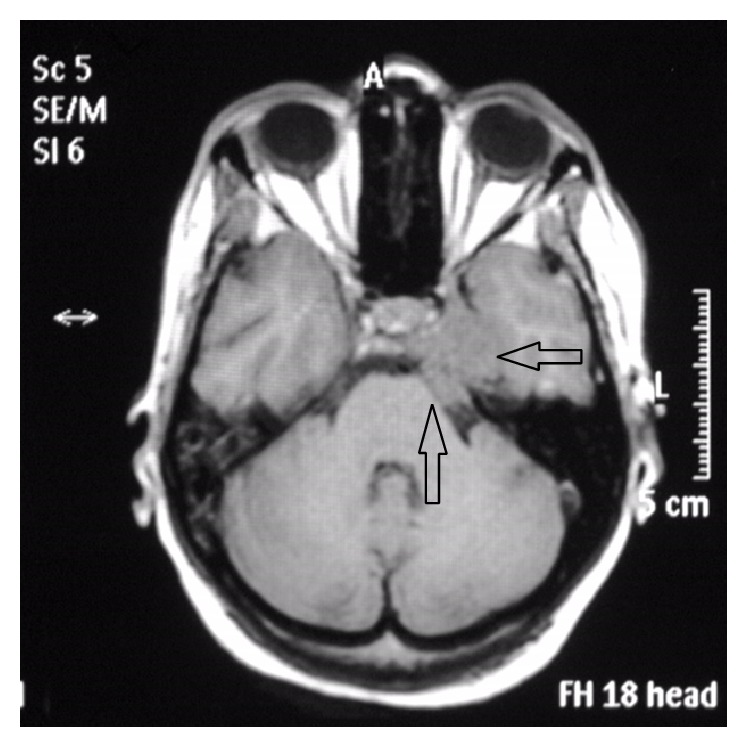
MRI precontrast showing altered signal intensity of the clivus, axial section that appears hyperintense.

**Figure 3 fig3:**
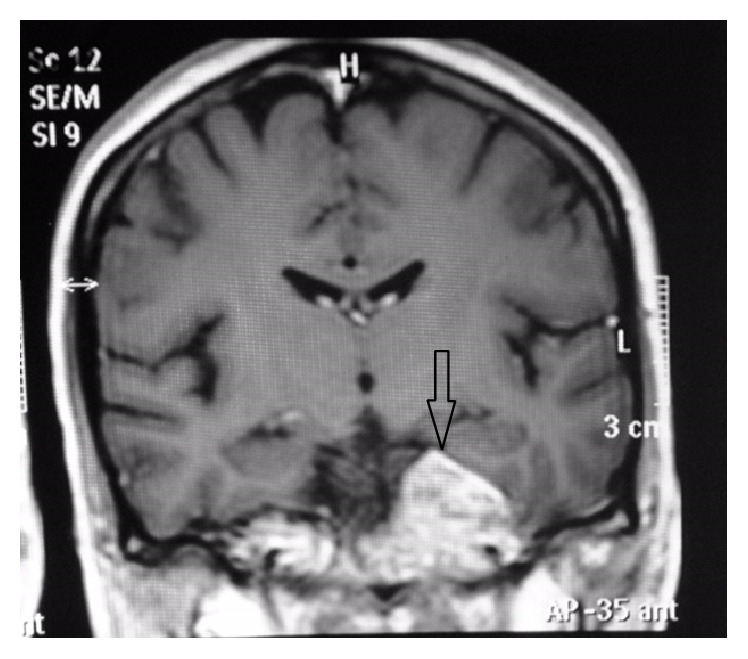
MRI precontrast showing altered signal intensity of the clivus, coronal section that appears hyperintense.

**Figure 4:**
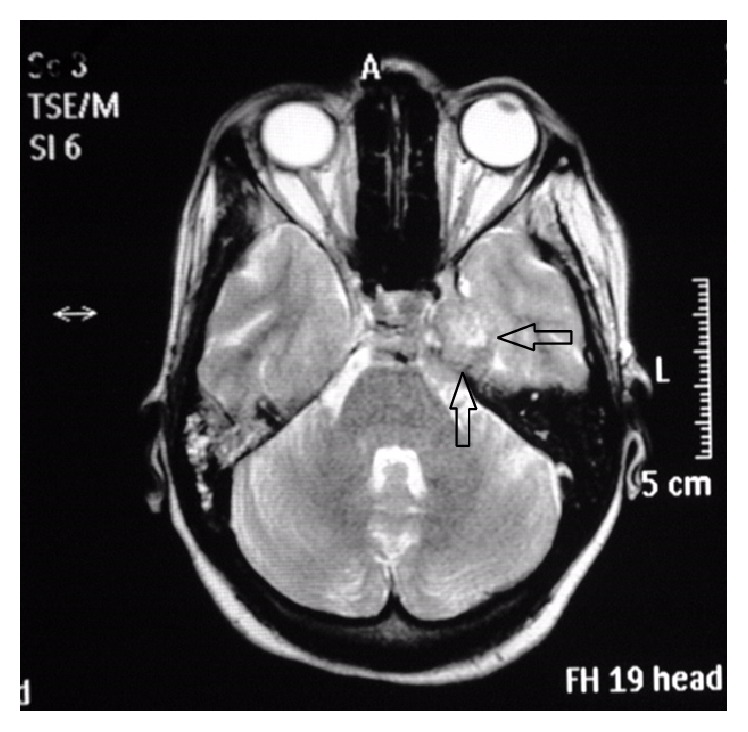
MRI postcontrast showing altered signal intensity of the clivus, axial section that appears heterogeneously hyperintense.

Thoracolumbar region MRI revealed a heterogeneously enhanced 5–7 cm solid renal mass suspicious of RCC. A large destructive mass noted to the right of the sacrum with extension into the right sciatic notch with a heterogeneous enhanced large left renal mass suspicious of RCC was also noted which was biopsied and pathology revealed metastatic poorly differentiated RCC with focal sarcomatoid features. MRI of the brain (Figures 1 [Fig fig2] [Fig fig3]–4) with contrast demonstrated altered signal intensity of the clivus on the left side, which gave an appearance of hyperintensity on T1-weighted images and heterogeneous hyperintensity with T2-weighted images. After contrast, there was marked enhancement of the lesion. The lesion approximately measured 3.6 cm × 3.3 cm × 3 cm. While there was no compression of the brainstem or pons, there was extension into the pontine cistern and bowing of the clivus. The lesion extended into the left cavernous sinus enlarging it anterosuperiorly. There was encasement of the carotid canal and left internal carotid artery resulting in narrowing. There was also extension into Meckel's cave. She was immediately given intravenous dexamethasone, which led to initial improvement of her symptoms. She was offered biopsy of the clivus mass, decompressive surgery that she refused because of her religious inclination, chemotherapy, and radiation therapy. She was subsequently planned for transfer to a nursing home after initial improvement in her symptoms.

## 3. Discussion

The most accurate incidence of RCC with metastasis specifically to the clivus is unknown and reports are very rare with few cases being reported [1–5, 19]. RCC patients may present with hematuria, flank pain, and flank mass felt on palpation. Patients are usually elderly males who have a smoking history. An atypical presentation of RCC with metastasis to the clivus is typical of RCC, a tumor that displays inconsistent and multifaceted symptoms and signs [1–3]. Even though this patient did not present with symptoms and signs of metastasis to the clivus at the outset, reports suggest that RCC presents with metastases to the lungs, liver, brain, and bones in about 50% of patients [3].

Majority of the reports of metastases to the clivus have been reported in case reports, case images, or series [15]. See Table 1 summarizing specifically renal cell carcinoma cases with metastases to the clivus [4, 5] and Table 2 summarizing all clival metastases from different primary sites [5, 14–18]. Metastases to the clivus have arisen from sites such as the prostate, breast, and stomach [7, 18]. Late recurrence of cancer with metastasis to the clivus after a period of remission has been reported [13]. Clival metastasis is a rarity more common in middle-aged men.

It is imperative that we consider the anatomy of the clivus and the abducens nerve. The clivus is a surface of a segment of the occipital and sphenoid bones in the skull surrounded by neurovascular structures of the brainstem and both internal carotid arteries [18]. The abducens nerve arrives at the subarachnoid space after leaving the pons. In the subarachnoid space, it runs beside the clivus bone and arrives at Dorello's canal making it vulnerable to damage. It then arrives at the cavernous sinus where it is contiguous and medial with and to the internal carotid artery, respectively. It then arrives at the orbit via the superior orbital fissure [9, 16].

Symptoms of clival metastasis include initial metastasis in about 40% of patients presenting with malignancy for the first time; symptoms include cranial nerve pathology of the abducens nerve [1–4, 19]. Studies have shown that Valsalva movements and spread to the internal vertebral venous plexuses may be responsible for cancer spread from the pelvis [1, 5].

Findings of clival metastasis are not very specific even though cancer growth in the clivus would cause dramatic cranial nerve pathologies and pain considering the unique course of the abducens nerve from the brainstem to the superior orbital fissure; it is highly susceptible to damage [1] and sometimes there is extension to other cranial nerves. Prognosis of patients with clival metastasis is not encouraging; median survival varies between 2 and 3 years [10]. In the event that there is cranial nerve palsy, prognosis becomes worse and surgery may not improve prognosis [18]. Radiological investigations such as CT scan and MRI are useful. CT scan assesses the degree of bone damage or calcification while MRI assesses the structures in the posterior fossa [18, 20]. Treatment varies based on symptoms and locations of the mass, or the histology of the primary cancer; it is either by radiotherapy which is the standard, resection of the mass endoscopically if the tumor is operable, or chemotherapy or both radiotherapy and chemotherapy [1–5, 10, 17, 19].

## 4. Conclusion

RCC with spread to the clivus is rare; it is important to have a high index of suspicion of RCC because of its unusual presentation. A symptom of diplopia may be the only presenting complaint in such patient. If the diagnosis is missed, it may lead to significant morbidity and mortality.

## Figures and Tables

**Table 1:** Cases of clivus metastasis specifically from RCC.

Author	Year	Age	Sex	Presentation	Treatment
Fumino et al. [4]	1998	58	Male	Diplopia	Radiotherapy, left nephrectomy
Mendelson et al. [5]	2014	59	Female	Diplopia	Endoscopic endonasal skull base surgery
Okudo et al. (present case)	2016	54	Female	Diplopia	Radiotherapy and palliative care

**Table 2:** Cases of clival metastasis available from PubMed (and other databases) along with the primary and presentation.

Author(s)	Year of reporting	Primary	Presentation
Turner et al. [6]	1980	Ewing's sarcoma (femur)	Diplopia
Fumino et al. [4]	1998	Renal cell carcinoma	Diplopia
Ulubas et al. [7]	2005	Lung cancer (SCC)	Headache
Escarda et al. [8]	2006	Hepatocellular carcinoma	Diplopia
Malloy [9]	2007	Prostate cancer (adenocarcinoma)	Diplopia
Marchese-Ragona et al. [10]	2008	Cancer of the tonsil (SCC)	Diplopia
Pallini et al. (case series) [2]	2009	Lung cancer (adenocarcinoma) (*n* = 2) Lung cancer (SCC) (*n* = 1) Prostate cancer (adenocarcinoma) (*n* = 2) Melanoma (skin) (*n* = 1)	Diplopia
Kolias et al. [11]	2010	Prostate cancer (adenocarcinoma)	Multiple cranial neuropathy
Ng et al. [12]	2011	Breast cancer	Ophthalmoplegia
Fukushima et al. [13]	2012	Stomach cancer (signet ring cell carcinoma)	Headache and diplopia
Bohnstedt et al. [14]	2012	Soft tissue myoepithelium (left hip)	Ophthalmoplegia
Kendre et al. [15]	2014	Rectal carcinoma	Diplopia
Mendelson et al. [5]	2015	Renal cell cancer	Diplopia
Kapoor et al. [16]	2015	Breast cancer	Diplopia
Rao et al. [17]	2015	Cervical cancer	Headache and vomiting
Lee et al. [18]	2015	Gastroadenocarcinoma	Diplopia
Okudo et al. (present case)	2016	Renal cell carcinoma	Diplopia

SCC: squamous cell carcinoma.

This table was adapted with permission from Kapoor et al. [16].

## References

[1] DeConde A., Sanaiha Y., Suh J. D., Bhuta S., Bergsneider M., Wang M. B. (2013). Metastatic disease to the clivus mimicking clival chordomas. *Journal of Neurological Surgery Part B: Skull Base*.

[2] Pallini R., Sabatino G., Doglietto F., Lauretti L., Fernandez E., Maira G. (2009). Clivus metastases: report of seven patients and literature review. *Acta Neurochirurgica*.

[3] Patel A. A., Kuperan A. B., Liu J. K., Patel C. R., Sharer L. R., Eloy J. A. Renal cell carcinoma with metastasis to the clivus.

[4] Fumino M., Matsuura H., Hayashi N., Arima K., Yanagawa M., Kawamura J. (1998). A case of renal cell carcinoma with metastasis in clivus presenting as diplopia. *Hinyokika Kiyo*.

[5] Mendelson Z. S., Patel A. A., Eloy J. A., Liu J. K. (2015). Endoscopic palliative decompression of the cavernous sinus in a rare case of a metastatic renal cell carcinoma to the clivus. *British Journal of Neurosurgery*.

[6] Turner J. L., Sweeney P., Hardy R. (1980). Ewing's tumor metastatic to the clivus, simulating meningitis: case report. *Neurosurgery*.

[7] Ulubas B., Ozcan C., Aydn O., Saritas E. (2005). Clivus metastasis of a squamous cell carcinoma: a rare location. *Journal of Clinical Neuroscience*.

[8] Escarda A., Vaquer P., Bonet L., Miralbés S., Gómez C., Obrador A. (2006). Clivus metastasis from hepatocarcinoma associated with transarterial hepatic chemoembolization. *Gastroenterologia y Hepatologia*.

[9] Malloy K. A. (2007). Prostate cancer metastasis to clivus causing cranial nerve VI palsy. *Optometry*.

[10] Marchese-Ragona R., Maria Ferraro S., Marioni G. (2008). Abducent nerve paralysis: first clinical sign of clivus metastasis from tonsillar carcinoma. *Acta Oto-Laryngologica*.

[11] Kolias A. G., Derham C., Mankad K. (2010). Multiple cranial neuropathy as the initial presentation of metastatic prostate adenocarcinoma: case report and review of literature. *Acta Neurochirurgica (Wien)*.

[12] Ng E. S., Tan S. H., Ling W. H., Venkatesh S. K., Wong C. I. (2011). Ophthalmoplegia in a patient with breast cancer. *Annals of the Academy of Medicine, Singapore*.

[13] Fukushima M., Katayama Y., Shigemori Y., Miyake H., Hirayama T., Kotani A. (2012). Clivus metastasis from gastric signet ring cell carcinoma after a 10-year disease-free interval. *Neurologia Medico-Chirurgica*.

[14] Bohnstedt B. N., Tomcik M., Eads T., Hagen M. C., Shah M. (2012). Metastasis of soft-tissue myoepithelial carcinoma to clivus. *Journal of Neurosurgery: Pediatrics*.

[15] Kendre B., Deopujari C., Karmarkar V., Ratha V. (2014). A rare case of carcinoma rectum metastasing to clivus. *Neurology India*.

[16] Kapoor A., Beniwal V., Beniwal S., Mathur H., Kumar H. S. (2015). Isolated clival metastasis as the cause of abducens nerve palsy in a patient of breast carcinoma: a rare case report. *Indian Journal of Ophthalmology*.

[17] Rao A. S., Nandennavar M., Narayanan G. S. (2015). Carcinoma cervix presenting with clivus metastasis. *Journal of Cancer Research and Therapeutics*.

[18] Lee A., Chang K., Hong H., Kim H. (2015). Sixth cranial nerve palsy caused by gastric adenocarcinoma metastasis to the clivus. *Journal of Korean Neurosurgical Society*.

[19] Sagoh M., Murakami K.-I., Oizumi T. (1996). Skull base metastasis from renal cell carcinoma presenting as abducens nerve paresis: report of two cases. *Neurological Surgery*.

[20] Wang A., Kleinman G., Murali R., Wainwright J., Tandon A. (2015). Metastatic renal cell carcinoma mimicking trigeminal schwannoma in a patient presenting with trigeminal neuralgia. *Journal of Neurological Surgery Reports*.

